# Does weighted vest use during weight loss influence long-term weight loss maintenance? A pilot study in older adults living with obesity and osteoarthritis

**DOI:** 10.1038/s41366-025-01795-5

**Published:** 2025-05-11

**Authors:** Carson DeLong, Barbara J. Nicklas, Daniel P. Beavers, Jason Fanning, Kristen M. Beavers

**Affiliations:** 1 Departments of Health and Exercise Science, Winston-Salem, NC USA; 2 Departments of Internal Medicine, Section on Gerontology and Geriatric Medicine, Wake Forest University School of Medicine, Winston-Salem, NC USA; 3 Departments of Statistical Sciences, Wake Forest University, Winston-Salem, NC USA

**Keywords:** Medical research, Randomized controlled trials

## Abstract

The purpose of this study was to explore whether and how gravitational loading during intentional weight loss (WL) influences subsequent weight regain. Pilot data come from a convenience sample of 18 older adults (70.4 ± 3.1 years, 83% women, 78% white) with obesity who participated in a 6-month WL intervention and also returned for 24-month follow-up assessment. Participants were originally assigned to 6-months of caloric restriction plus 10 h/day weighted vest use (WL+VEST; *n* = 9) or caloric restriction only (WL Only; *n* = 9). Body weight (BW) and resting metabolic rate (RMR) were collected at baseline, 6, and 24 months. WL+VEST and WL Only participants lost significant and similar amounts of BW by 6-months [WL+VEST: –11.2 kg (95% CI: −14.6, −7.7) versus WL Only: –10.3 kg (95% CI: −13.7, −6.8)]; *p* = 0.71. By 24-months, the WL+VEST group regained approximately half of lost BW [−4.8 kg from baseline (95% CI: −9.6, 0.1)], while the WL Only group regained all lost BW [+0.9 kg from baseline (95% CI: −3.9, 5.8)]; *p* = 0.10. Change in RMR from baseline to 6 months was −16.3 (95% CI: –100.8, 68.2) kcal/day and −237.5 (95% CI: −321.9, −153.0) kcal/day for the WL+VEST and WL Only groups, respectively (*p* < 0.01); and was modestly and inversely associated with change in BW from 6 to 24 months (r = −0.39, *p* = 0.11). Pilot data signal weighted vest use during caloric restriction may be associated with reduced weight regain via preserved RMR.

**Figure:**
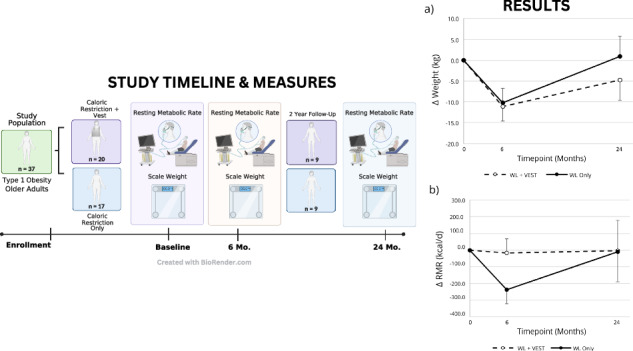
Study Overview and Results. Created with BioRender.com.

Study Overview and Results. Created with BioRender.com.

## Introduction

Obesity is a serious, common, and costly public health problem—particularly among older adults [1]. Lifestyle-based weight loss (WL) interventions are currently first-line therapies for obesity management, with data from our group and others demonstrating 8–10% WL over one year is an achievable target for this population [2, 3]. Long-term WL maintenance, however, remains elusive for most [4].

Well-known biological adaptations to WL that predispose individuals to weight regain include decreased resting and non-resting energy expenditure, as well as increased hunger cues via changes in appetite-regulating hormones [5]. Recent data also point to changes in gravitational loading as a potential driver of weight regulation. Coined the “gravitostat” in 2018, this intriguing hypothesis posits that lower extremity osteocytes sense changes in body weight (BW) and respond by sending a systemic signal to the brain, influencing appetite and subsequent body fat stores [6]. To date, this hypothesis has been largely tested in murine models [7–9]; however, a landmark study in humans demonstrates that increased gravitational loading produces small—but significant—weight and fat mass loss over a 3-week period [10]. Further confirmation of this compelling hypothesis could influence current weight management practices.

The purpose of this pilot study is to contribute to this active area of research by utilizing follow-up data from the Arthritis Pilot for Preserving Muscle While Losing Weight (NCT02239939) study to explore whether and how weighted vest use during intentional weight loss influences long-term WL maintenance among older adults living with overweight and obesity.

## Materials/subjects and methods

### Study overview

Full methodologic details, including inclusion/exclusion criteria, of the original study (NCT02239939) can be found in the published primary outcome paper [11]. Briefly, this randomized controlled trial (RCT) was designed to assess the feasibility of 6-months of daily use of a progressively weighted vest (as an alternative to traditional exercise) during WL in older adults with obesity and self-reported arthritis (WL+VEST; *n* = 20), as compared to WL Only (*n* = 17), and to explore treatment effect estimates on several measures of musculoskeletal health. Subsequently, a convenience sample of 18 participants (*n* = 9/group) who completed the 6-month trial voluntarily returned for a 24-month long-term follow-up assessment (average follow-up duration: 25.4 months) and represent the study sample for the present analysis (see Supplementary Fig. 1).

### Intervention descriptions

During the 6-month intervention period, all participants were instructed to follow a dietary WL intervention without a formal exercise program. Calorie deficit was achieved via the Medifast^®^ 4 & 2 & 1 Plan^®^, estimated to provide 1100–1300 kcal/day. This meal plan included a total of 4 meal replacement products, with the addition of 2 lean and green meals and 1 healthy snack. Weekly group nutrition/behavioral counseling sessions led by a Registered Dietitian were also incorporated. The dietitian guided participants on their food choices and portion sizes and encouraged participants to consume only what was approved as a part of the meal plan. Participants randomized to the weighted vest group (WL+VEST) additionally received an appropriately sized vest (Hyper Vest PRO®, Austin, TX), based on their ability to wear the vest under clothing and complete a full range of motion/chest expansion without restriction, and were asked to wear the vest up to 10 h/day during the most active part of their day (with a goal of at least 50% of awake time wearing the weighted vest). The weight of the vest was increased weekly by study staff, with the goal of replacing all lost weight up to a maximum amount of 15% of the participant’s baseline weight. Compliance with the weighted vest protocol (including weight in vest and wear time) was collected weekly via self-report. Participants were asked to follow the dietary prescription and wear the vest during the first 6-months, only; participants were not contacted or on study protocol during the year after the intervention ended.

### Demographic and outcome assessments

Demographic characteristics (age, sex, race) and height (Heightronic 235D stadiometer, QuickMedical, Issaquah, WA) were collected at baseline. All outcome assessments were collected at baseline, 6 and 24-months by trained and blinded assessors. Body mass (Detecto scale, Detecto, Webb City, MO) was measured without shoes or outer garments and resting metabolic rate (RMR) was measured using an Ultima CCM™ Indirect Calorimetry system in the morning after a 12-hour fast and absence from exercise during the prior day. Finally, total body fat and lean masses were obtained from whole-body DXA scans using a Delphi QDR; Hologic (Marlborough, MA).

### Statistical analyses

Baseline characteristics were summarized overall and by randomized treatment group as means and standard deviations (mean ± SD) for continuous variables or counts and percentages [*n* (%)] for discrete variables. BW, lean mass, and RMR change estimates come from a mixed model containing treatment group, visit, treatment group by visit interaction, and baseline measure of the outcome and presented as means and 95% confidence intervals (95% CI). Pearson correlation (r) was used to determine associations between change in RMR during the WL period and change in BW during the follow-up period. Analyses were performed using SAS v.9.4 (SAS Institute Inc. Cary, NC) software, with *p*-values of 0.05 used to determine statistical significance.

## Results

### Participant baseline characteristics and weighted vest compliance

Baseline demographic and clinical characteristics of participants who returned for the 24-month assessment visit (*n* = 18) are presented by group in Supplementary Table 1, and did not differ materially from those who did not (*n* = 19; see Supplementary Table 2). Overall, participants were 70.4 ± 3.1 years of age, 83.3% female, and 77.8% Caucasian. Average BMI was 35.2 ± 2.8 kg/m^2^ and RMR was 1397.8 ± 241.2 kcal/day. Participants in the WL+VEST group (*n* = 9) wore the vest for an average of 6.6 ± 2.2 h/day with the weight in the vest averaging 6.1 ± 2.0 kg of baseline BW, which is similar to what was previously reported for the full study sample [11].

### Change in body weight, lean mass, and RMR by group and time

**Fig. 1:**
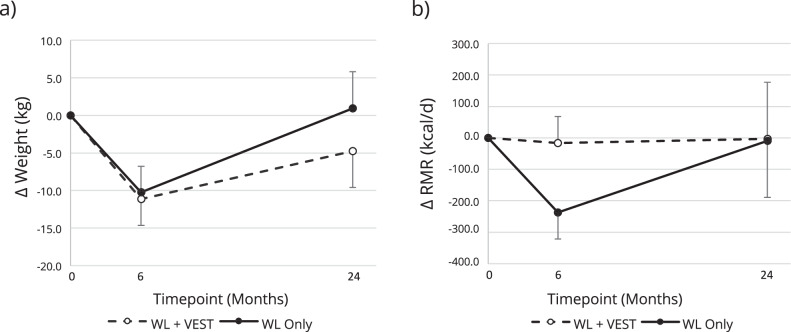
Change in body weight and resting metabolic rate by group and time. **a** Change in weight (kg) at three timepoints: baseline, 6, and 24 months, where open circles with the dashed line represent the WL+VEST group and closed circles with the solid line represent the WL only group. **b** Change in resting metabolic rate (kcal/d) at three timepoints: baseline, 6, and 24 months, where open circles with the dashed line represent the WL+VEST group and closed circles with the solid line represent the WL only group.

Change in BW by group over time is shown in Fig. 1a. On average, WL+VEST and WL Only participants lost significant and similar amounts of weight during the active 6-month WL intervention [WL+VEST: −11.2 (95% CI: −14.6, −7.7) kg versus WL Only: −10.3 (95% CI: −13.7, −6.8) kg]; between group *p* = 0.71, of which about 1/4^th^ was lean mass loss [WL+VEST: −3.0 kg (95% CI: −4.0, −1.9) versus WL Only: −2.8 kg (95% CI: −3.8, −1.8)]; between group *p* = 0.82. By 24-months, the WL+VEST group regained approximately half of lost weight [−4.8 kg from baseline (95% CI: −9.6, 0.1) kg], while the WL Only group regained all lost weight [+0.9 kg from baseline (95% CI: −3.9, 5.8)]; between group *p* = 0.10. Likewise, by 24-months, lean mass was still significantly reduced in WL+VEST group [−1.84 kg from baseline (95% CI: −3.4, −0.3)] while lean mass in the WL only group returned to baseline [−0.4 kg from baseline (95% CI: −2.0, 1.2)]; between group *p* = 0.19.

**Fig. 2:**
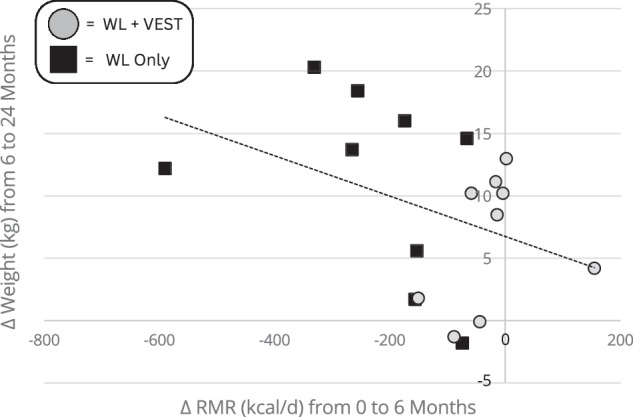
Scatterplot of change in RMR from 0–6 months versus change in weight from 6 to 24 Months. Gray circles represent the WL+VEST group participants, black squares represent the WL only group participants.

Change in RMR by group over time is shown in Fig. 1b. Average 6-month RMR change was −16.3 (95% CI: −100.8, 68.2) kcal/day and −237.5 (95% CI: −321.9, −153.0) kcal/day for the WL+VEST and WL Only groups, respectively (between group *p* < 0.01). At 24-months, RMR returned to baseline for both groups [−3.5 kcal/d from baseline (95% CI: −183.7, 176.6) and −9.4 kcal/d from baseline (95% CI: −189.5, 170.8) for the WL+VEST and WL Only groups, respectively]; between group *p* = 0.96. Fig. 2 shows the moderate inverse correlation (r = −0.39, *p* = 0.11) observed between change in RMR during the active WL period (i.e. 0 – 6 months) and change in weight during the follow-up period (i.e. 6–24 months).

## Discussion

The primary purpose of this pilot study was to explore whether gravitational loading during intentional WL influences weight regain following treatment. We found that 24 months after randomization to a highly effective 6-month weight loss intervention, the WL Only group regained nearly twice as much weight as the WL+VEST group. Additionally, and intriguingly, concomitant use of a weighted vest abolished the reduction in RMR often otherwise seen during caloric restriction [12]. Further, change in RMR during active WL was modestly and inversely associated with weight change during the follow-up period.

Data from this pilot study complement and extend prior knowledge examining the role of gravitational loading as a potential homeostatic regulator of weight change. Given the innovative nature of this topic, much of the existing data come from preclinical models, where “loading” is achieved by surgically implanting weighted pellets into mice and results in significant WL [6–9]. In contrast to these studies, we did not observe a differential effect of gravitational loading on achieved WL, which may be due to the specificity/rigidity of our 6-month dietary prescription. However, we did observe a signal for differential weight regain in the free-living follow-up period. This finding is in alignment with results from the Jansson 2018 [6] and 2021 [9] publications, where mice randomized to sustained loading (i.e. heavy capsule followed by heavy capsule) experienced sustained BW and fat mass loss compared with mice randomized to removal of the load (i.e. heavy capsule followed by empty capsule).

To our knowledge, only one other study has examined the influence of gravitational loading on weight change in humans [10]. In this seminal trial, Ohlsson and colleagues randomized 72 middle-aged adults living with obesity to wear heavily loaded (11% BW; *n* = 36) versus lightly loaded (1% BW; *n* = 33) weighted vests for 8 h/day over a 3-week period. In agreement with murine model data, the high load treatment resulted in greater WL compared to low load treatment [mean difference: −1.37% (95% CI: −1.96, −0.79)], although it is important to note that this degree of WL is less than what would be considered clinically meaningful [13]. Work from our project extends these findings by examining legacy effects of gravitational loading on weight change, while also adding preserved RMR during active weight loss as a potential explanatory variable for successful WL maintenance.

Major strengths of this study include the novelty of the weighted vest intervention, the RCT design, and the long period of intervention (6-months) and follow-up (24-months). That said, inferential ability of this pilot is limited by its size and scope, and future work should aim to validate these findings in a larger and more diverse sample, and to better elucidate the effect of gravitational loading on additional drivers of weight change — particularly those pre-specified in the gravitostat theory — including osteocyte signaling and appetite hormones. Additionally, our method for collecting 24-month follow-up data relied on a convenience sample, which may have resulted in biased estimates; however, we did use ANCOVA methods and adjusted for baseline values of each outcome to minimize this possibility. Finally, we suggest future studies collect more detailed information regarding protocol compliance, including weighted vest wear time spent in a standing position versus seated position as well as dietary intake data.

In sum, results from this pilot study suggest that among older adults who wore a weighted vest during caloric restriction, initial WL was better preserved at 24-months, which may be due to preserved RMR. Future work should aim to confirm these findings in an adequately powered trial.

## Supplementary information

Supplementary Tables 1 & 2

Study CONSORT Diagram
