# Supplementary material for: Does weighted vest use during weight loss influence long-term weight loss maintenance? A pilot study in older adults living with obesity and osteoarthritis
Source: Int J Obes (Lond). 2025 May 11;49(8):1662–5. doi: 10.1038/s41366-025-01795-5 (PMC12396959; doi:10.1038/s41366-025-01795-5)
Supplement: Supplementary file 1 — Supplementary Tables 1 & 2 [file 41366_2025_1795_MOESM1_ESM.docx]

**Supplementary Table 1**. Baseline Demographic and Clinical Characteristics of 24-Month Follow-Up Study Sample (n=18).

| Characteristics | WL Only (n=9) | WL+VEST (n=9) |
| --- | --- | --- |
| Age (years) | 69.8 ± 2.5 | 71.1 ± 3.6 |
| Gender, n (%) |  |  |
| Male | 0 (0) | 3 (33) |
| Female | 9 (100) | 6 (67) |
| Race, n (%) |  |  |
| White/Caucasian | 6 (67) | 8 (89) |
| Multi-racial or Other | 3 (33) | 1 (11) |
| Body Mass (kg) | 93.6 ± 14.1 | 100.0 ± 8.8 |
| Body Mass Index (kg/m^2^) | 35.3 ± 2.9 | 35.0 ± 2.9 |
| Total Body Fat Mass (kg) | 45.3 ± 6.5 | 45.9 ± 9.6 |
| Total Body Lean Mass (kg) | 44.3 ± 7.8 | 49.4 ± 5.9 |
| Resting Metabolic Rate (kcal/d) | 1365.6 ± 304.9 | 1430.1 ± 168.2 |

Continuous data are presented as means (standard deviations) and categorical data are presented as absolute numbers (percentage).

**Supplementary Table 2**. Baseline Demographic and Clinical Characteristics of Participants from Original Pilot Trial who did not Return for the 24-Month Assessment Visit (n=19).

| Characteristics | WL Only (n=8) | WL+VEST (n=11) |
| --- | --- | --- |
| Age (years) | 70.0 ± 2.8 | 69.6 ± 3.3 |
| Gender, n (%) |  |  |
| Male | 3 (37) | 3 (18) |
| Female | 5 (63) | 9 (82) |
| Race, n (%) |  |  |
| White/Caucasian | 6 (75) | 8 (73) |
| Multi-racial or Other | 2 (25) | 3 (27) |
| Body Mass (kg) | 94.4 ± 9.2 | 98.5 ± 12.1 |
| Body Mass Index (kg/m^2^) | 35.5 ± 3.4 | 35.5 ± 2.9 |
| Total Body Fat Mass (kg) | 42.0 ± 7.8 | 45.7 ± 8.7 |
| Total Body Lean Mass (kg) | 47.9 ±8.8 | 48.3 ± 10.3 |
| Resting Metabolic Rate (kcal/d) | 1595.3 ± 338.5 | 1255.5 ± 265.9 |

Continuous data are presented as means (standard deviations) and categorical data are presented as absolute numbers (percentage).
