# Supplementary figures and images for: Does weighted vest use during weight loss influence long-term weight loss maintenance? A pilot study in older adults living with obesity and osteoarthritis
Source: Int J Obes (Lond). 2025 May 11;49(8):1662–5. doi: 10.1038/s41366-025-01795-5 (PMC12396959; doi:10.1038/s41366-025-01795-5)

**Supplementary Figure 1. Study CONSORT diagram**

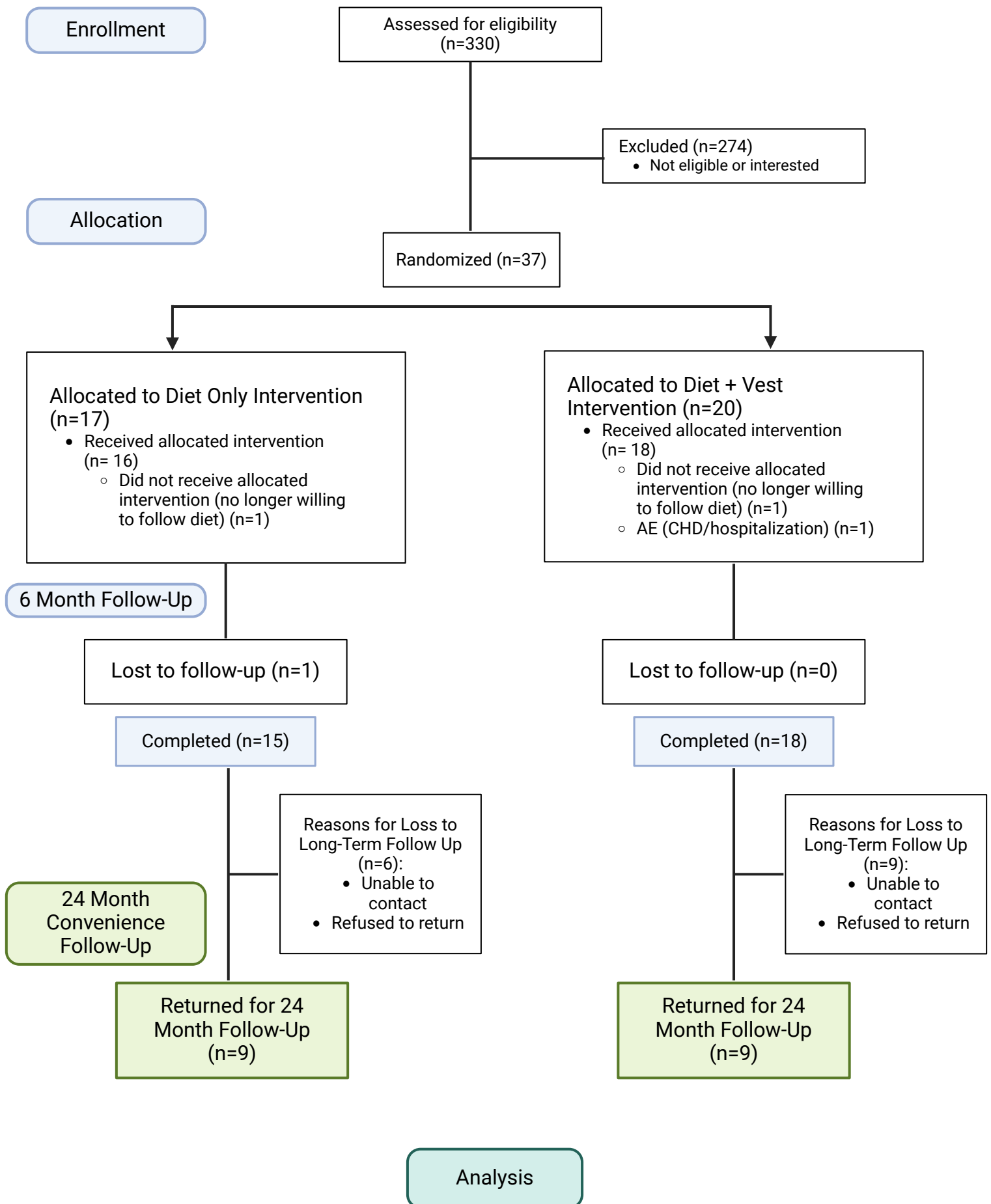

Supplement: Supplementary file 2 — Study CONSORT Diagram [file 41366_2025_1795_MOESM2_ESM.pdf]
